# The South African Competition Commission COVID-19 easing of competition rules for private healthcare to facilitate public-private interaction - a media content analysis

**DOI:** 10.1186/s12913-024-11994-x

**Published:** 2024-12-02

**Authors:** Amina Abdullah, Thatohatsi Sefuthi, Mapato Ramokgopa, Sharon Fonn, Lungiswa Nkonki

**Affiliations:** 1 Department of Global Health, Division of Health Systems and Public Health, Stellenbosch University, Cape Town, South Africa; 2 School of Public Health, University of the Witwatersrand, Johannesburg, South Africa; 3 School of Public Health and Community Medicine, University of Gothenburg, Gothenburg, Sweden; 4 Faculty of Medicine and Health Sciences, Walter Sisulu University, Mthatha, South Africa

**Keywords:** COVID-19, Competition policy, Block exemption, Media, Content analysis, Public health emergency response and preparedness

## Abstract

**Background:**

Future emergencies from climate degradation or diseases are likely, prompting ongoing investment in emergency readiness and learning from country responses. South Africa’s healthcare system, divided into public and private sectors, required a coordinated, intersectoral response to the COVID-19 pandemic. A coordinated action that the South African government took was adapting competition regulations. The South African Department of Trade, Industry and Competition on 19 March 2020, published a block exemption (easing of competition rules) for healthcare to promote standardization of practices across the health sector and facilitate agreements between the National Department of Health and the private sector to ensure adequate service delivery to all South Africans.

**Methods:**

We assessed how much coverage the block exemption for healthcare received in the media and if the scope included details of what the exemption meant, how stakeholders and the public could use it, and the resulting public sentiment. We conducted a media content analysis to select, screen and assess the media material. Online and social-media articles in the public domain from 19 March 2020 to 19 March 2021, in English were considered.

**Results:**

We analysed 22 online media articles that matched our inclusion criteria. Twenty of these reflected a positive sentiment, and two were negative. Media reported on the COVID-19 block exemption in healthcare only in the first three months of our study period. The articles primarily communicated what the block exemption meant, focusing on allowing collaboration, the purpose of the exemption, the activities granted under the exemption and the actors to whom it applied. It’s estimated that these media articles could have been read by up to 432,003 people out of a total population of just under 43 million people over the age of 15 years.

**Conclusion:**

In times of crisis, the media has a significant responsibility to provide accurate information to the public. However, the accuracy and reliability of such information depends on the quality of official sources on which the media relies. Our research shows that very few media reports were available about the block exemption for healthcare. While the government implemented the exemption, it did not communicate its purpose directly to the public. Our research highlights the need for better communication between policymakers and the media.

## Background

Within the first three months of 2020, the novel coronavirus developed into a global public-health emergency [1]. On 11 March 2020, the World Health Organization (WHO) officially declared it a pandemic. Governments worldwide needed to respond rapidly to the COVID-19 pandemic [2].

South Africa, an upper middle-income country [3], had an estimated population of 59,62 million in 2020 [4]. The healthcare system comprises public and private sectors [5]. The majority of the South African population, 84%, access health services through the tax-funded public sector and 16% of the population buy voluntary private medical insurance [6]. South Africa’s healthcare system faces challenges related to uneven coverage and access to funding, poor infrastructure, and human-resource constraints [7]. For example, there are currently 814 hospitals in the country, with 405 in the public sector and 409 in the private sector [7]. Before the pandemic, in 2019, the national density of medical specialists was 16.5 per 100,000 people. However, there was a significant difference between the number of specialists working in the public sector, at only 7 per 100,000, and the private sector, at 69 per 100,000 population [8].

The COVID-19 pandemic exposed the vast inequality in access to care, in particular, specialist care in the South African healthcare system [9]. Before the pandemic, the public healthcare system was already under pressure due to shortages of staff and resources [10]. The pandemic required the country to work together and ensure adequate healthcare delivery, and highlighted the need for a significantly more integrated healthcare response [11]. The pandemic led to an overwhelming increase in demand for healthcare services, putting a strain on the capacity of healthcare workers to deliver effective care and ensure suitably equipped health facilities [11].

Neither the already overstretched public nor the private healthcare system was likely to survive the demands of the outbreak on its own [6]. Containing and preventing the spread of COVID-19 needed coordinated, intersectoral action. One such action was that the South African government exempted specific stakeholders in the healthcare sector from certain regulations of the Competition Act.

The Competition Commission is a South African statutory body established under the Competition Act and has the authority to investigate, regulate and assess restrictive business practices [12]. The Commission aims to promote equity and efficiency in the economy and competition regulations seek to create an environment where consumer products and services are at competitive prices, and ethical business conduct sustains the economy [11].

Thus, the Department of Trade, Industry and Competition (DTIC), which also received a request from and consulted with some private-sector players, exempted the healthcare sector from the application of Sects. 4 and 5 of the Competition Act during the pandemic and termed this a block exemption which was published in the Government Gazette on 19 March 2020. It aimed to promote the standardization of practices across the health sector and facilitate agreements between the National Department of Health (NDoH) and the private sector to ensure an adequate and coordinated COVID-19 healthcare response for all South Africans [11]. Ordinarily, competing private-sector firms are legally not allowed to coordinate as this would be regarded as collusion in terms of Sect. 4 of the Competition Act [13]. The block exemption meant that hospitals and healthcare facilities, medical suppliers, medical specialists and radiologists, pathologists and laboratories, pharmacies, healthcare funders, and the public-healthcare sector were exempted from abiding by Sects. 4 and 5 of the Competition Act and could meet collectively to promote access to healthcare, prevent exploitation of patients, enable the sharing of healthcare facilities, manage capacity and reduce prices.

The announcement of the COVID-19 block exemption in healthcare was not the only response to the COVID-19 health crisis [14], in addition the DTIC issued the Consumer and Customer Protection and National Disaster Management Regulations and Directive, to protect consumers from possible exploitation of market power enabled by the crisis.

Gupta and Sinha argue that the media is a social institution that supports the establishment of dominant discourses [15]. Thus, the media influences how people understand the world around them and how they behave [16, 17]. Chouliaraki and Fairclough describe discourses as “diverse representations of social life which tend to simplify complex realities and are inherently positioned by different social actors who see and represent social life in different ways” [18]. Meaning is socially constructed and consistent with the constructivist paradigm [19]. Thus, discourses in media reflect and influence public perception [20].

The media plays a vital role in the health system [21], because it educates the public about health and well-being, health policy and its implementation or lack thereof [22], and regulation changes. Media is also a means by which the public can be made aware of how government actions and policies affect them, and the government, in turn, can gain feedback on their activities and procedures [21].

Media studies that have been conducted in the health policy and systems research field have been restricted to high-income countries and need to be more inclusive of the media representation of health-policy processes [20].

In a study of print-media coverage of primary healthcare and related research evidence in South Africa, the authors concluded that media does not report on every health issue; instead, media organizations prioritise items for publication based on what they consider newsworthy and, as a result, the information covered is selective [23].

Furthermore, the use of social media as a tool to support public-health initiatives has been identified. For example, during the listeriosis outbreak in South Africa during 2017–2018, guided toolkits were transcribed into the major South African languages and shared from many locations and social-media platforms [24]. Health professionals, the United States’ Center for Disease Control and Prevention (CDC), and health activists shared prevention measures and related outbreak information via social media. Social media has therefore created a platform for the health system and the public to engage. In a review of the challenges and opportunities of social media as a tool for communication, Kubheka et al., 2020 suggested that social-media platforms are essential agents for information exchange. However, they need to be utilized more, given their potential to enable access to real-time, credible information [24]. More research is needed on the role of media in communicating South African health policy [23].

Given the restrictions on movement and gatherings during hard lockdowns, media was the primary communication source during the COVID-19 pandemic [25] and the media played a significant role in connecting policymakers, health professionals, and the public [26]. As such media could affect pandemic outcomes due to the variety of media sources, (online, print and broadcast media being examples), broad public reach [25] and coverage.

It is, therefore, useful to understand if a system response like the block exemption was communicated and the extent of that coverage.

While there is generally some coverage of health issues in the media, for instance, coverage of advertising of sugar-sweetened beverages and around gender-based violence, this study assessed media coverage of a policy issue, emanating outside of the health sector but impacting on the health sector (the DTIC), using media content analysis. The four other South African media studies we identified covered varied health issues ranging from microbicide trials for HIV, of International Nurses’ Day, National Health Insurance (NHI), and student suicide [20, 27–29]. There is still a need to understand mainstream media as a method to communicate with health-system actors. There also appears to be a gap in the extent of media coverage about health-policy issues and further research should be done to explore the role of all media (radio, TV, social, print, etc.) in communicating health issues and to better understand the media’s role in shaping the health-policy process [20].

In this project, we investigated how a change in competition regulation of private healthcare during a state of emergency was communicated in the media.

## Methods

We conducted a media content analysis which is a specialized sub-set of content analysis and adopted its approach for selecting, screening and assessing the media material. We chose this approach as it is a systematic method to study mass media and describe who said what, through which channel, to whom, and with what effect [30].

Our search terms included: Competition Act Block Exemption, Competition Law Block Exemption, Competition healthcare block exemption, Healthcare, COVID-19, Private health response, South Africa, Universal Health Coverage, Department of Trade, Industry and Competition, National Department of Health, National Health Insurance and COVID-19, Private healthcare, Public Healthcare, Contracting with Private healthcare, Private Hospital groups, Medical Schemes, Private healthcare practitioners, PPE procurement, vaccine procurement.

Online media and two social-media platforms, Twitter (now X) and Facebook, were searched for content relating to the block exemption. All online media articles and social-media content available in English from 19 March 2020 to 19 March 2021, were collected for inclusion.

The search was subcontracted to Meltwater, an online media monitoring company experienced in academic and non-academic content exploration. The research team had a workshop with Meltwater where the project rationale was explained, and preliminary search terms and media domains were provided.

The pilot revealed that a strict inclusion criterion that demanded all the terms be present in the title (rather than and / or) would result in missing relevant media listings because several online articles discussed the block exemption even though there was no mention of it in the title. For instance, included article number 2, “African competition authorities respond to the Covid-19 crises”, only mentions COVID-19. Another example is Article 5 (See Table 1), which does not include healthcare, COVID-19, or block exemption in the title “Commission to deal with suppliers who inflate prices”. Thus, after discussions with Meltwater and considering the observations, it was decided that all media mentioning at least one search term would be included to avoid missing relevant articles.

**Table 1:** Retrieved articles reporting on the COVID-19 block exemption into healthcare

Number	Headline	Publication Date
1	Covid-19: Competition& consumer law developments affect consumer & healthcare sectors	23-Mar-20
2	African competition authorities respond to the COVID-19 crisis	25-Mar-20
3	BHF’s ‘great concern’ over CMS circular on medical aid member support and exemption guidelines	29-Jul-20
4	Block Exemption from provisions of the Competition	01-Apr-20
5	Commission to deal with suppliers who inflate prices	20-Mar-20
6	Competition Commission still seeing price hikes up to 900%vduring coronavirus outbreak	19-May-20
7	Competition law exemptions and regulations applicable during Covid-19	26-Mar-20
8	Covid-19 and competition law: clothes, coffee shops, care services & more	31-Mar-20
9	Covid-19: Block exemption regulations for shopping mall Tenants and Landlords	30-Mar-20
10	Covid-19: Commission to deal with suppliers who inflate prices	23-Mar-20
11	SPOTLIGHT OP-ED: Covid-19: Zweli Mkhize is responsible for coordinating with private sector	05-Apr-20
12	African competition authorities respond to COVID- 19 crises	07-Apr-20
13	Competition Act 89 of 1998 – COVID-19 Block Exemption for the Healthcare Sector, 2020 (GNR 349) added to our website	25-Mar-20
14	DTI effects regulations for healthcare sector, retailers in light of Covid-19	20-Mar-20
15	MEDIA STATEMENT: Civil society organisations call for principled contracting with private sector in the COVID-19 health response, as well as more coordination and transparency	21-May-20
16	Minister Ebrahim Patel on Covid-19 National Disaster Regulations	20-Mar-20
17	Minister has exempted agreements or practices from restrictions on competitors and customers/suppliers in the Competition Act	20-Mar-20
18	Any developments on the payment holidays and other interventions by the banks to alleviate hardship on citizens during #COVID19SouthAfrica?	24-Mar-20
19	OPINION | How exemptions to the Competition Act help the fight against coronavirus	27-Mar-20
20	South Africa: Competition Law Exemptions and Regulations applicable during COVID-19	24-Mar-20
21	South Africa: COVID-19 - Minister Mkhize Is Responsible for Coordinating with Private Sector	03-Apr-20
22	Covid-19: Competition & consumer law developments affect consumer & healthcare sectors	20-Mar-20

The initial search yielded a total of 8877 media content listings. Relevant information was extracted from the content into spreadsheets and included the date, headline, URL, opening text, source, and online reach. Articles published online typically have a like or dislike button and we extracted the sentiment from these using the Meltwater platform. Two reviewers (AA and TS) independently assessed the identified content. Headlines were first evaluated for relevance to the search terms, after which both reviewers met to discuss any discrepancies. Thereafter both reviewers assessed the media listings content over several weeks for relevance to the COVID-19 block exemption into healthcare. The reviewers (AA and TS) resolved discrepancies through discussions; if AA and TS could not reach a consensus, they contacted senior researcher LN. Most of the included media material did not refer to the block exemption into healthcare and was therefore excluded for further analysis. Three senior researchers (LN, SF, and MR) conducted a quality assessment on 19 of the articles found to be discrepant between AA and TS as an additional quality assurance process. All duplicate media listings and reposts were excluded. Overall, 22 articles/posts were included and are presented in Table 1.

### Inclusion criteria

Articles written in English, posted between 19 March2020and 19 March 2021, with content referring to the COVID-19 block exemption into healthcare.

### Exclusion criteria

Written in any language other than English, posted before 19 March 2020 and after 19 March 2021, and content not descriptive of the COVID-19 block exemption into healthcare.

**Fig. 1:**
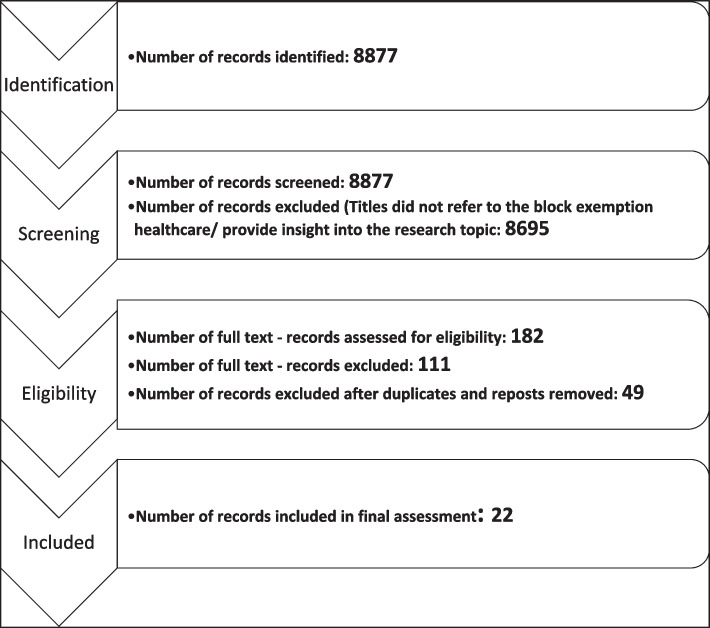
Prisma flow diagram

Reasons for exclusion are provided in the PRISMA flow diagram (Fig. 1).

#### Data extraction and analysis

We developed a data-extraction template and piloted it on three articles. We extracted the following information from the included articles: Publication details, what was said about the block exemption, if the meaning of the block exemption and its uses were made clear and what the public sentiment was as assessed using the online like or dislike button. Two authors screened the titles of the media listings that were generated for inclusion after which full texts were reviewed. Two authors extracted and coded data independently. A first set of codes were assigned. The data were read again, and a second set of codes assigned, and then checked for consistency, with conflicts resolved through discussion and consensus reaching. The study did not make use of intercoder reliability as we were interested in the diverse perspectives of multiple researchers, and thus it was not appropriate for our study [31]. Codes were generated using an inductive thematic analysis approach. AA and LN read the extracted data a third time in line with the emergent themes listed on a separate document to verify between both reviewers and finalize with a third reviewer.

## Results

We analysed the 22 included media articles that matched our inclusion criteria. We found that 20 of the included articles received a positive sentiment and only two a negative sentiment from the public. The estimated reach of these media indicated that 432,003 people could have read the articles. This was estimated by noting the number of views associated with each post. Eight key themes emerged from the coding process. These are (1) Period during which the exemption will be effective; (2) Purpose; (3) Practices not exempted; (4) Examples of how the block exemption should be used in practice; (5) Actors and activities exempted by the block exemption into healthcare; (6) Reporting requirements; (7) Unified health response; and (8), Call for transparency in the process, all of which are elaborated upon below. Overall, the content covered in the articles varied − 16 covered only some of the eight themes. Six articles covered the block exemption into healthcare in their title, but the content did not include any descriptive information that fell within our analysis (A3, A4, A8, A9, A17, A18).

### Themes

1. Period during which the exemption will be effective

Seven of the included articles reported the effective date of the block exemption as “19 March 2020”, and four stated when the exemption would expire. The block exemption was only in place while the pandemic remained a national disaster.

2. Purpose

Twelve articles explained the purpose of the block exemption.

#### Collaboration

Eleven of the 12 articles discussed collaboration as the primary purpose of the block exemption. Some news reports (A1, A5, A10, A16) described the cooperation to be between private health sector players:

*“These regulations will permit private healthcare providers to coordinate their actions as part of the National Department Health efforts*,* including sharing facilities and beds*,* medical supplies*,* nurses and doctors between different companies and the Government.” (A16)*.

While others (A2, A7, A15, A11, A19) interpreted the block exemption to include both public and private health sectors:

*“…The block exemption regulations allow unprecedented coordination between public and private health institutions*,* including hospital groups and private sector professionals*,* to deliver equitable health services. These attempts are still unclear (A15).”*

Three articles (A1, A19, A21) referred to coordinating and preventing harm to the patient (including harm in the form of price hikes):

*“Regulation 3 allows various private healthcare sector players to enter into agreements or practices for the sole purpose of*:

*Coordination of patient*,* service*,* and healthcare worker allocations between private facilities and procurement of consumables and other inputs required for optimal patient treatment - this should result in you being able to access health care once you’ve been referred to a facility.” (A21)*.

*“… addresses competition and consumer protection concerns that came about due to COVID-19” “The regulations were published*,* to allow for consumer concerns around price hikes of essential items” (A1)*.

Nine articles referred to the Competition Act and explained what was permitted under the block exemption that would otherwise not be allowed in the absence of the exemption:

*“… several players in the sector will not be in contravention of the Competition Act if they have to coordinate an emergency response” (A19).*

#### Ensuring adequate supply-side response

Several articles regarded sufficient private healthcare response to the pandemic as another purpose of the block exemption (A6, A7, A19).

*“…according to regulation 4*,* these agreements are permitted to make available additional capacity at private healthcare facilities to the public healthcare sector and to ensure adequate medical supplies to the public health sector.” (A19)*.

*“The regulations also allow agreements and practices between the private healthcare sector and the DoH to support the DoH” (A14*,* A21).*

*“According to regulation four*,* these agreements are permitted to make additional capacity available at private healthcare facilities to the public healthcare sector and to ensure adequate medical supplies to the public healthcare sector.” (A21)*.

*“…to alleviate*,* contain and minimise the effects of the national disaster*,* and to promote access to healthcare…” (A14)*.

3. Practices not exempted

Five articles mentioned what was not included in the exemption (A5, A7, A10, A14, A19, A22).

*“The Block Exemption only applies if the agreement or practice is undertaken at the request of*,* and in coordination with*,* the Department of Health (DOH) to respond to the COVID-19 pandemic national disaster. The exemption excludes communication and agreements regarding prices unless authorised by the Minister of Health.” (A22)*.

4. Examples of how the block exemption should be used in practice

Some articles provided examples of how private-health players cooperated with the public sector, while others observed the need for more transparency around the agreements and reporting requirements (A5, A19).

*“Some private health sector actors have availed themselves and indicated their readiness to collaborate with the public health sector” (A21).*

*“Netcare had committed to treating public patients in Netcare facilities on a not-for-profit cost recovery basis. However*,* Netcare will assess and pre-authorise any referral from the public sector on a case-by-case basis” (A21).*

5. Actors and activities exempted by the block exemption into healthcare

Several newspaper articles listed private healthcare players to which the block exemption applies, hospitals (A1, 14, A16, A19, A20, A22), healthcare facilities (A1, A14, A16, A20, A22), medical suppliers (A14, A16, A20), medical specialists and radiologists (A14, A16, A20), pathologists and laboratories (A14, A16, A19, A20), pharmacies (A14, A16, A19, A20), healthcare funders (A14, A16, A20), medical aid funds (A19). Some described how the actors were supposed to use the exemption.

*“ healthcare funders and healthcare facilities*,* as well as other healthcare providers*,* with the sole purpose of reducing the cost of diagnosis*,* tests and diagnostics*,* treatment*,* and other preventative measures” (A1*,* A22)*.

Several articles identified the DOH as the steward of this block exemption into healthcare.

*“The cooperation and coordination need to be at the request of the DoH.” (A10)*.

6. Reporting requirements

One article reported the reporting requirements for the role players participating in the block exemption (A22).

*“All relevant players in the healthcare sector who participate in any agreements or practices falling within the scope of the Block Exemption must keep minutes of meetings held and written records of such agreements or practices.” (A22)*.

7. Unified health response

Three articles conveyed the block exemption as an act of unity and highlighted the need for a unified health system in South Africa (A15, A19, A21).

*“…these regulations acknowledge the need for close collaboration and coordination to ensure access to healthcare*,* prevent the exploitation of patients and provide an essential public good*,* guaranteed in Sect. 27 of the Constitution.” (A21)*.

8. Call for transparency in the process

Only one article (A15) called on the DoH and the National Corona-Virus Command Council to embark on a transparent and principled contracting process with the private sector.

*“Engagement with the private health sector has happened outside the public eye. Contracting with the private sector for medical goods and services is unclear. There is not yet a comprehensive approach to these agreements.” (A15)*.

## Discussion

We identified eight key themes. These communicated what the block exemption meant with a focus on promoting collaboration, the purpose of the exemption, the activities allowed under the exemption, and the actors to whom it applied.

We concluded that there were limited media reports about the block exemption for healthcare overall and a lack of comprehensive reporting. There was no direct communication with the public on how the easing of the competition regulation would benefit the South African population. We found that only one media article called for transparency in the contracting process with the private sector. Furthermore, there was no additional media content on the block exemption practices after the first and second COVID-19 waves, which were characterized by rapid spread in infection, hospital admissions, and increased mortality rates [32], and during the vaccine rollout.

A study that explored newspaper coverage of COVID-19 in South Africa found that media coverage was predominantly sensationalist and that most media did not provide health information [33]. We found this to be true in general but less so around the technical issue of the block exemption. Facts about the block exemption were thin and vital information about the exemption practices was not communicated via the media. For instance, explaining how the exemption sought to find a balance between adequate response to the pandemic but still protecting consumers and alerting consumers to be aware that price gouging was still not allowed. Another study that investigated online news headlines of the COVID-19 pandemic in South Africa, the USA, and Italy, found that headlines did cover “government response” to the pandemic [34], as is the case here. Many of the online articles covered the block exemption for healthcare by headline only but lacked supporting information within the narrative.

The role of the media is to be a ‘watchdog’ for policy implementation, especially during the pandemic when movement was restricted. This ensures that the government’s actions are transparent and accountable. When there are communication gaps between the government and the media, it can have several implications. Inaccurate and untimely reporting can lead to public misunderstanding of guidelines, regulations and available resources which can potentially worsen the public health crisis [35]. It can also reduce public trust in the government. Poor communication between the government and the media can also disproportionately impact marginalized communities, particularly those with existing barriers to accessing healthcare resources, as they may remain uninformed about available support [36].

Our findings align with the speculation made by Akintola et al. [23] that the media does not necessarily report on every matter affecting a health issue; rather, the media prioritizes subjects for publication based on what they consider newsworthy. Thus, it is government’s responsibility to engage the media strategically during times of crisis. In fact the WHO recommends that authorities should promptly engage mass media to communicate important and necessary information about managing epidemics and pandemics [37].We have also found no other study investigating media coverage of the block exemption for the healthcare sector. In March 2021, the Competition Commission of South Africa published a report on the impact of the exemption assessed until October 2020 and confirmed the positive effect of the exemption [14]. This report is the only publicly available document sharing detailed insights into the block exemption.

We are likely to see other emergencies in the future, which may result from climate degradation or another infectious viral diseases [38]. Thus, international organizations are continuing to invest in emergency readiness and preparedness. We suggest that guidelines may assist in ensuring sufficient and accurate communication between government and the media so that reporting on crucial policy changes during international and national emergencies is achieved.

We found that the media reported that the block exemption was allowed, and the activities exempted, however, detailed reports on the exempted activities and partnerships or the outcomes thereof were not reported.

The results raise the question of whether the media was invited to meetings on the block exemption and whether the government communicated adequately with the media regarding the policy.

These findings suggest an opportunity for further research into the determinants restraining or facilitating media coverage of health-policy issues in South Africa and suggest that further opportunities should be taken to understand better how policymakers engage with mass media.

We recommend including journalists on international and national emergency teams and that guidelines should be developed to assist how governments and journalists communicate during emergencies. Furthermore, journalists could play a greater oversight role and not only write about the change in regulations or policy but also follow up on the implementation or lack of implementation of policy changes during emergencies. Transparency is essential to building trust. Regular press briefings to the media could have ensured that more information about the block exemption (easing of competition regulations) was shared with the public.

Media reporting could have played a helpful role by clarifying that the exemption aimed to balance an adequate pandemic response with consumer protection, including a reminder that exploitative conduct such as price gouging remained prohibited despite the exemption. Such coverage might have encouraged the public to report sudden price increases, including by the media, where such conduct occurred. Changes in competition regulation was a technical process during COVID-19 pandemic. It was reported often in part and usually verbatim in the news articles we reviewed. Little opportunity to subvert the purpose or content of the exemption thus occurred – misinformation was not spread. However, there was little analysis by journalists of the exemption and the potential use or misuse of the exemption.

This study had limitations. South Africa recognizes twelve official languages. In our study, we only included media articles published in English. At the onset of the pandemic, communication about policies was mainly in English. We also performed this media analysis on online news and social-media platforms, which may be a study limitation. Print media was excluded from this study due to several media firms having to close their doors during the lockdown period. Information was therefore shared via online and social-media platforms. Radio was also excluded. This was not a big limitation as the actors affected by the block exemption were most likely to have access to and read print media.

## Conclusion

This was the first study to systematically examine media reporting on the relaxation of competition policy to aid universal coverage of the health-system response to the COVID-19 pandemic. Previous research has shown that media are pivotal in informing the public about crucial information during a health crisis. Yet, the media can only report on a pandemic insofar as it is shared from official sources. Our analysis verifies that most of the information reported was similar across the included articles and was published in the first quarter of the pandemic. This is an important lesson. We need more robust communication between policymakers and the media to report on policy issues. This evidence can be used to guide key health informants and journalists to improve information exchange and writing on public health and health systems in times of crisis. Insights gained from our study are transferrable to other settings and can be adopted by the global community to leverage the power of media in planning for future crises or pandemics and throughout health policy-making processes. We recommend the inclusion of media representatives to the country emergency preparedness teams to streamline communication strategies during crises. This integration allows journalists to understand better the unique communication needs of emergencies, distinct from non-emergency contexts. Such a collaboration would equip media professionals to effectively convey urgent behavioural guidelines and policy changes that may benefit the health system and the population which may not require direct public action. Additionally, preparedness training should include scenario-based communication planning to anticipate diverse messaging needs, mitigate misinformation risk and ensure consistent public understanding. These strategies can help bridge the gap between research, media, and policy. Researchers and health experts should improve collaboration with journalists and actively participate in information dissemination. Additionally, governments can support public interest journalism, while individuals should be encouraged to become more critical consumers of health information [39]. Media firms should not only focus on informing the public of health policy but rather report on health policy in a manner that can ignite political and public debate.

## Data Availability

The data generated and analysed for this study can be made available by the authors upon request.

## Abbreviations

HREC: Health Research Ethics Committee
NDoH: National Department of Health
SU: Stellenbosch University
USA: United States of America
WHO: World Health Organization
CDC: Centres for Disease Control and Prevention
